# Supramolecular Polymer Nanocomposites for Biomedical Applications

**DOI:** 10.3390/polym13040513

**Published:** 2021-02-09

**Authors:** Xiumei Li, Wanjia Xu, Yue Xin, Jiawei Yuan, Yuancheng Ji, Shengnan Chu, Junqiu Liu, Quan Luo

**Affiliations:** 1 State Key Laboratory of Supramolecular Structure and Materials, College of Chemistry, Jilin University, 2699 Qianjin Street, Changchun 130012, China; lixiumei_chem@163.com (X.L.); xuwj1997@163.com (W.X.); xinymavis@gmail.com (Y.X.); yuanjw1318@mails.jlu.edu.cn (J.Y.); jiyc1216@gmail.com (Y.J.); 15732622036@163.com (S.C.); junqiuliu@jlu.edu.cn (J.L.); 2 College of Material, Chemistry and Chemical Engineering, Hangzhou Normal University, Hangzhou 311121, China; 3 Key Laboratory of Emergency and Trauma, Ministry of Education, College of Emergency and Trauma, Hainan Medical University, Haikou 571199, China

**Keywords:** supramolecular polymer nanocomposite, biomedical application, therapeutic delivery, bioimaging, tissue engineering

## Abstract

Polymer nanocomposites, a class of innovative materials formed by polymer matrixes and nanoscaled fillers (e.g., carbon-based nanomaterials, inorganic/semiconductor nanoparticles, metal/metal-oxide nanoparticles, polymeric nanostructures, etc.), display enhanced mechanical, optoelectrical, magnetic, catalytic, and bio-related characteristics, thereby finding a wide range of applications in the biomedical field. In particular, the concept of supramolecular chemistry has been introduced into polymer nanocomposites, which creates myriad “smart” biomedical materials with unique physicochemical properties and dynamic tunable structures in response to diverse external stimuli. This review aims to provide an overview of the chemical composition, morphological structures, biological functionalities, and reinforced performances of supramolecular polymer nanocomposites. Additionally, recent advances in biomedical applications such as therapeutic delivery, bioimaging, and tissue engineering are also discussed, especially their excellent properties leveraged in the development of multifunctional intelligent biomedical materials.

## 1. Introduction

With the rapid development of materials science, higher requirements are being put forward for the structure, property, and functional diversity of polymer materials. Additionally, there is a growing emphasis on the personalized design of materials to meet special requirements, especially in the field of biomedicine. Polymer nanocomposites, as a class of innovative materials, attract considerable attention because of their programmable functions and outstanding properties [1,2]. From the perspective of chemical composition, polymer nanocomposites are matrix–filler combinations composed of polymer matrixes and nanoscaled reinforcing phases (either organic or inorganic constituents). Their performance is prominently dependent on the inherent properties of polymer matrixes, the reinforcing effects from nanofillers, and the production processes. The incorporation of organic or inorganic phases into polymer matrixes further upgrades their new properties such as enhanced mechanical, good optoelectrical, magnetic, catalytic, and bio-related characteristics [3,4,5]. By choosing appropriate nanofillers, loading amounts, and processing methods, it is even possible to obtain customized functional nanocomposites.

On the basis of specificity and reversibility, supramolecular chemistry provides the possibility of dynamic and intelligent control of polymer nanocomposites. Supramolecular polymer nanocomposites, which perfectly combine the advanced properties of polymer nanocomposites with the advantages of supramolecular chemistry, hold great promise as a novel class of multifunctional “smart” materials. Unlike covalent polymer nanocomposites, supramolecular polymers nanocomposites are totally or partially governed by various supramolecular interactions, including hydrogen bonding, metal coordination, π–π interactions, host–guest interactions, and electrostatic interactions. Basically, supramolecular interactions are incorporated into each stage of polymer nanocomposites preparation such as polymer interconnections, the coupling between polymer matrix and nanofiller, or even both. The intrinsic directivity and reversibility of supramolecular interactions not only allow efficient access to molecular order and morphology control, but also endow supramolecular polymer nanocomposites with fascinating dynamic structures in response to diverse external stimuli (e.g., such as pH, temperature, ionic strength, light, voltage, etc.) [6,7,8,9,10]. These features are absolutely attractive and desired by materials science, thus paving the way for a wide range of applications. In general, supramolecular polymer nanocomposites integrate the advantages of dynamics, stability, and versatility, which may inspire future revolutions in many fields, especially biomedicine.

In this review, we summarize the chemical composition, biomedically relevant properties, and versatile applications of several representative supramolecular polymer nanocomposites in terms of their structure-property relationship. In particular, we highlight how scientists reinforce material performance through rational design, thereby leveraging their various applications in biomedical field including therapeutic delivery, bioimaging, and tissue engineering.

## 2. Classification of Supramolecular Polymer Nanocomposites

Typically, supramolecular polymer nanocomposites can be categorized into three main types according to the filler constituents (carbons, inorganic/semiconductor, metal/metal oxide). We will discuss some examples below, where different functional nanomaterials are integrated into a polymer matrix to improve stability and mechanical strength, or even give brand-new properties.

### 2.1. Carbon-Based Supramolecular Polymer Nanocomposites

**Figure 1:**
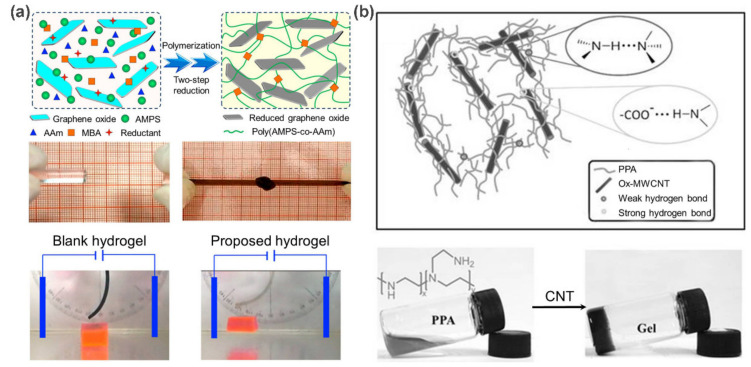
(**a**) Schematic representation of the fabrication of rGO/poly(AMPS-*co*-AAm) nanocomposite hydrogels. Photographs show their improved mechanical properties and obvious electro-responsive bending behaviors. (**b**) The formation of ox-MWCNTs/PPA hydrogels via hierarchical hydrogen bonds. Adapted with permission from Refs. [15,16].

Carbon-based nanomaterials, including 0D fullerenes, 1D carbon nanotubes (CNTs), 2D graphene, etc., represent an important group of nanofillers. The prominent mechanical properties, and electrical and thermal conductivities make them ideal functional additives to incorporate multifunctionality into polymers. Typically, the inclusion of CNTs or graphene can significantly improve the mechanical, electrical, and thermal properties of the final materials [11,12,13], thereby showing a broad application prospect. For example, Tang et al. [14] developed a single-wall carbon nanotubes (SWCNTs)-reinforced supramolecular polymer hydrogel via in situ free radical polymerization. Owing to the reinforcement effect of the SWCNTs, the resulting nanocomposites showed both an improved tensile strength (over 300%) and a faster electromechanical response capability. Similar enhancements were also observed in graphene reinforced polymer composites. Chu and co-workers [15] fabricated a novel electro-responsive nanocomposite polymer hydrogels by incorporating the reduced graphene oxide (rGO) nanosheets into poly(2-acrylamido-2-methylpropane sulfonic acid-coacrylamide) (poly(AMPS–*co*–AAm)) networks via two-step reduction (Figure 1a). As a result, both the tensile strength and compressive strength of the nanocomposite hydrogels are remarkably improved by the hydrogen bond networks between rGO nanosheets and poly(AMPS–*co*–AAm) chains. Moreover, the inherent electronic conductivity of rGO nanosheets further endows these nanocomposite hydrogels with excellent electro-responsive properties for possible applications as “soft cantilevers” and “soft grippers”.

Besides the above improvements in mechanical and electrical performances, CNTs and graphene can also work as crosslinkers to guide the formation of supramolecular carbon-based polymer nanocomposites through non-covalent interactions. Especially for oxidized CNTs (ox-CNTs) and graphene oxide (GO), the introduction of various amphiphilic oxygen-containing groups (carbonyl, carboxyl, hydroxyl, and epoxide) on the surface of CNTs and graphene not only facilitates their dispersion in aqueous media, but also allows for the noncovalent interactions with polymer chains through hydrogen bonding or van der Waals force, thus paving the way for the preparation of supramolecular polymeric nanocomposites. Accordingly, carbon-based supramolecular polymer nanocomposites have been widely used to engineer self-healing and stimuli-responsive materials. For example, Zhang et al. [16] fabricated a multifunctional nanocomposite hydrogel based on oxidized multi-walled carbon nanotubes (ox-MWCNTs) and polyethylene polyamine (PPA), where hierarchical hydrogen bonds (the combination of hydrogen bonds with different strength) act as ingenious driving forces for the hydrogel preparation and functional design (Figure 1b). As expected, the resultant ox-MWCNTs/PPA hydrogels integrate thermal responsiveness, pH responsiveness, and self-repair properties due to the dynamic and stimulus-responsive nature of the hydrogen bond network. On the other hand, attributing the unique photothermal response property of ox-MWCNTs, a fast gel–sol transition could be triggered by near-infrared (NIR) light. In this case, the gel could transform to the solution within 2 min upon exposure to NIR light, and then back to the gel state within 3 min under visible light. Similar self-repair behavior was also observed by Yan et al. [17] on chitosan/GO hydrogels. These features are particularly desirable for biomedical applications, such as tissue engineering and drug delivery, depicting a bright application prospect.

In addition, a supercritical CO_2_ (SC-CO_2_)-assisted process is able to reduce and exfoliate GO powders to improve their electrochemical properties. Sarno M. et al. systematically studied the effect of SC-CO_2_ processing on GO [18] and subsequently applied this process to the preparation of polymer/GO-based supercapacitor electrodes [19]. At the optimized GO loading (60% *w*/*w*), the supercapacitor reaches a very high energy density of 79.2 Wh kg^−1^ at a power density of 234 W kg^−1^ and exhibits excellent electrochemical properties.

### 2.2. Inorganic/Semiconductor Nanoparticle-Based Supramolecular Polymer Nanocomposites

**Figure 2:**
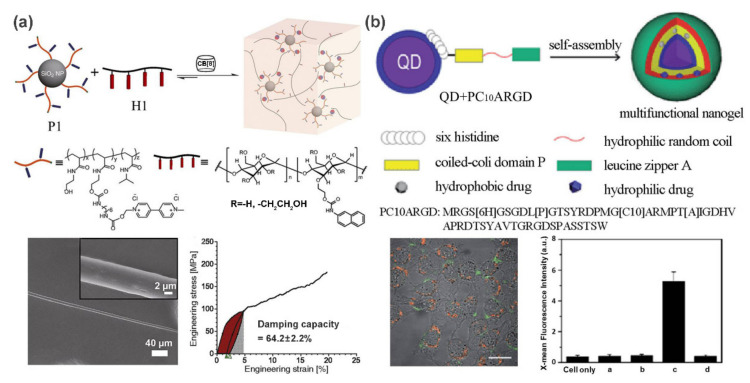
(**a**) CB [8]-mediated self-assembly of MV-functionalized silica nanoparticles and linear semicrystalline polymer into supramolecular nanocomposite hydrogel. Supramolecular fibers can be drawn from this hydrogel and exhibit a high damping capacity. (**b**) Schematic illustration of the formation process of QD/PC_10_ARGD nanogel. Confocal fluorescence image and flow cytometry analysis show its targeted imaging capable. Adapted with permission from Refs. [20,25].

Some inorganic nanoparticles, such as silica nanoparticles, mesoporous silica nanoparticles (MSNs), nanoclays, calcium phosphates, and bioactive glasses, play an important role in the mechanical and functional reinforcement of polymer materials. They have motivated extensive research interest due to their excellent biocompatibility, unique bioactive properties, and high mechanical and thermal stability [5]. Recently, Scherman et al. [20] developed a supramolecular fiber with excellent tensile and damping properties from a SiO_2_ nanoparticle-reinforced polymer hydrogel. As shown in Figure 2a, the hydrogels contain two main components: methyl viologen (MV)-functionalized polymer-grafted silica nanoparticles (P1) and a linear semicrystalline polymer H1 (hydroxyethyl cellulose functionalized with naphthalene (Np) isocyanate). Cucurbit[8]uril (CB[8]) acts as a crosslinker to trigger the self-assembly for the formation of stable and dynamic 3D network via host-guest interactions. Attractively, uniform supramolecular fibers that exhibit an extremely high damping capacity of 64.2 ± 2.2% and remarkable tensile properties can be readily drawn from this hydrogel to compete with viscose, artificial silks, and hair.

Similarly, clays are introduced as physical crosslinking points to improve the physical and mechanical properties of polymeric matrix. As a typical example, Aida and co-workers [21] developed a high-water-content (96%–98% water) hydrogel through the self-assembly of sodium polyacrylate (ASAP) pretreated clay nanosheets (CNSs) and dendritic molecular binders (Gn-binders, *n* = 1–3) decorated with multiple guanidinium ions at the ends of dendrites. CNSs are first electrostatically wrapped by ASAP to facilitate their dispersion in water. Subsequently, the cationic guanidine group quickly adheres to the anionic surface of CNSs to form mechanically tough (G′ up to 0.5 MPa) and transparent hydrogel. Additionally, the hydrogel displays self-healing behavior, as the assembly is driven by non-covalent interactions, which provides many possible applications, such as transporting biological activities. Beyond these improvements, these inorganic nanoparticles are also noted for fascinating bioactive properties. Calcium phosphates and bioactive glasses are frequently used to develop nanocomposite bioscaffolds with excellent mechanical and biological properties for tissue engineering applications [22,23].

Semiconductor quantum dots (QDs) possess several attractive advantages such as broad excitation and narrow emission spectra, high quantum yield, and unique size-dependent emission [24], making them ideal fluorescent additive for polymer matrix. In these cases, polymers exhibit rare optical properties, thereby opening up a new avenue for their applications in optical imaging. For example, a QD-reinforced polypeptide hybrid nanogel was fabricated by Liu and co-workers for targeted imaging [25]. By incorporating multiple functional domains into a polymer chain, a coiled-coil polypeptide (PC_10_ARGD) was fabricated. As shown in Figure 2b, PC_10_ARGD could be attached to the surface of GSH-capped CdSe-ZnS QDs by metal coordination, leading to a sandwich hydrogel layer surrounding the QDs formed through the association of the coiled-coil P domain. The formation of nanogel not only decreases the cytotoxicity to both HeLa cells and NIH 3T3 cells, but also promotes the imaging specificity due to the targeting effects of the RGD motif.

### 2.3. Metal/Metal-Oxide Nanoparticle-Based Supramolecular Polymer Nanocomposites

Metal [26,27,28] (gold, silver, copper, and other noble metals) and metal-oxide [29] (such as Fe_2_O_3_, Fe_3_O_4_, Al_2_O_3_, ZnO, and TiO_2_) nanoparticles exhibit many novel properties different from conventional solids, which enable the fabrication of advanced functional materials for optoelectrical, magnetic, catalytic, and biomedical applications. In particular, a wide variety of high-performance supramolecular polymer nanocomposites can be developed by combining them with polymers. Such nanocomposites are commonly prepared in two approaches: in situ synthesis of these nanoparticles in the polymer networks, or embedding surface-functionalized nanoparticles into the polymer networks. This combination not only inhibits the oxidation and agglomeration of metal nanoparticles to fully exert their specific properties, but also reduces the consumption of metal (especially noble metal), thereby leading this become a hot spot of materials science.

**Figure 3:**
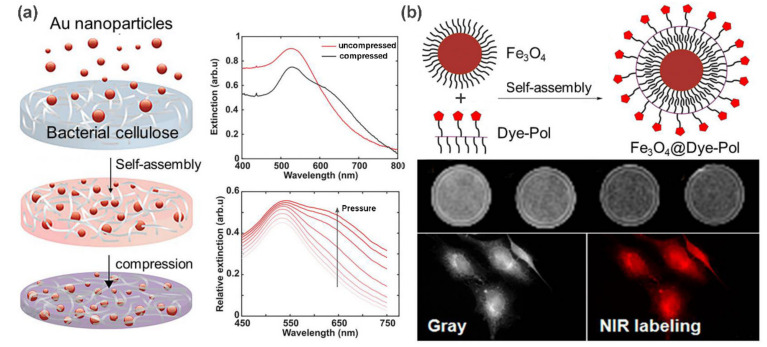
(**a**) Fabrication of mechanoplasmonic-responsive BC-AuNPs nanocomposite membranes. UV-vis spectra show the pressure-induced redshift in peak position. (**b**) Schematic illustration of the hybrid nanoparticle formation directed by hydrophobic interactions. MR imaging and NIR imaging results with Fe_3_O_4_@Dye-Pol. Adapted with permission from Refs. [31,33].

Among the above metal nanoparticles, gold nanoparticles (AuNPs) are noted for their unique optical and photothermal response properties, excellent electrical conductivity, and good biocompatibility. Their nanocomposites with polymer have been already used for diverse applications, including drug delivery, bioimaging, and tumor treatment [30]. In a recent example, Aili et al. [31] demonstrated the tunable functionalization of bacterial nanocellulose (BC) membranes with AuNPs through a self-assembly strategy. Large amounts of AuNPs were adsorbed and diffused into the interior of the BC membranes via electrostatic interactions to form red hybrid membranes as illustrated in Figure 3a. Interestingly, the resulting nanocomposites exhibited unique mechanoplasmonic properties. The pressure applied on the BC-AuNPs membranes results in tunable spectral variations and enhanced broadband absorption, which is attributed to the increase in the near-field coupling between the immobilized AuNPs. Not restricted to spherical AuNPs, this assembly strategy can be further extended to the loading of AgNPs, and anisotropic gold nanorods and nanoprisms. The resulting BC-metal NP nanocomposites are tailored for diverse properties such as antimicrobial properties, excellent senor performance, and tunable optical properties.

As a typical example of metal oxide nanoparticles, Fe_3_O_4_ nanoparticles have been extensively studied as a promising candidate for diverse applications, including magnetic resonance imaging (MRI), magnetic drug delivery, and catalysts [32]. Yen et al. [33] fabricated a self-assembly hybrid nanoparticle for dual-modal imaging by combining magnetic Fe_3_O_4_ nanoparticles and a NIR fluorescent dye (Figure 3b). It was demonstrated that Fe_3_O_4_@Dye-Pol displayed lower cytotoxicity than Fe_3_O_4_ nanoparticles, and could be used to label cancer cells for NIR fluorescence microscopy and enhance negative contrast for T2-weighted MR imaging. In addition, Fe_3_O_4_ nanoparticles can also be used to design shape memory materials to achieve a fast shape recovery under a magnetic field [34].

### 2.4. Other Nanoparticle/Nanostructure-Based Supramolecular Polymer Nanocomposites

Some other nanoparticles or nanostructures have also greatly enriched the types and functions of supramolecular polymer nanocomposites. For example, rare earth-doped upconversion nanoparticles (UCNPs) are another alternative fluorescent additive [35,36]. Their anti-Stokes shifts by near-infrared excitation are expected to make up for the deficiency of QDs, making their nanocomposites ideal candidates for in vivo fluorescence imaging probes. Metal-organic frameworks (MOFs) are known for their ultra-high porosity, as well as their tunable structure and functionality. Therefore, coating MOF nanoparticles with polymer network may generate reinforced drug delivery systems with specific performance [37]. Additionally, magnetic properties can be obtained when using Gd-MOF nanoparticles as an additive. The resulting nanocomposites have great potential as contrast agents of MRI [38]. In comparison to the above rigid nanomaterials, polymeric nano-assembly structures such as micelles, liposomes, and nanogels exhibit many desirable properties including good biocompatibility, physicochemical stability, biodegradability, and excellent flexibility. Because of their unique structures, they are also able to encapsulate hydrophilic or hydrophobic drugs, and thus are appropriate for developing drug delivery systems [39,40]. Some examples will be presented later in the therapeutic delivery section.

In general, supramolecular polymer nanocomposites exhibit many improved properties as compared to their components. As discussed above, these improvements are mainly reflected in three aspects: (1) the integration of the properties of each component; (2) the increased strength mainly attributed to the interfacial interactions between the polymer chains and the hybrid nanofillers; and (3) the dynamic and tunable structures mainly endowed by supramolecular interactions. In addition, multifunctional integration can be achieved easily through the rational integration of multiple reinforcing materials. Actually, the materials obtained in this way are currently more popular. They can better meet the requirements of complex production practices and have been widely used in various fields.

## 3. Biomedical Applications

A desirable biomedical material must have prominent biocompatibility, and highly tailored properties and functionalities to meet a wide variety of targeted biomedical needs. In this regard, supramolecular polymer nanocomposites have great potential as candidates for such applications. Polymers, especially those from natural origins, are known to have excellent biodegradability and biocompatibility, which are of great importance for in vivo applications. Furthermore, the stimuli-responsive capability, adequate mechanical strength as well as many special properties desired by biomedicine (e.g., optical, electrical, thermal, magnetic, catalytic, and antibacterial characteristics) can also be obtained when combining polymers with dynamic supramolecular interactions and diverse functional nanomaterials. In this section, we highlight the recent examples of these reinforced supramolecular polymer nanocomposites for biomedical applications, including therapeutic delivery, bioimaging, and tissue engineering.

### 3.1. Therapeutic Delivery

Supramolecular polymer nanocomposites have been widely used for therapeutic purposes in recent years. There are many advantages to using supramolecular polymer nanocomposites in cancer-related therapy and drug delivery. As drug carriers, they exhibit typical stimuli-responsive behavior and can serve as smart carriers to control the targeted and on-demand release of drugs by various internal and external stimuli (e.g., pH, light, redox agents, hot, electric field, enzyme, etc.) [41]. Besides the drug encapsulation and release, supramolecular polymer nanocomposites can also achieve multifunctional integration by combining multiple nanofillers. In this way, a variety of traditional and new emerging therapy strategies, such as chemotherapy, radiotherapy, photothermal therapy and photodynamic therapy can be integrated for combinational therapies.

**Figure 4:**
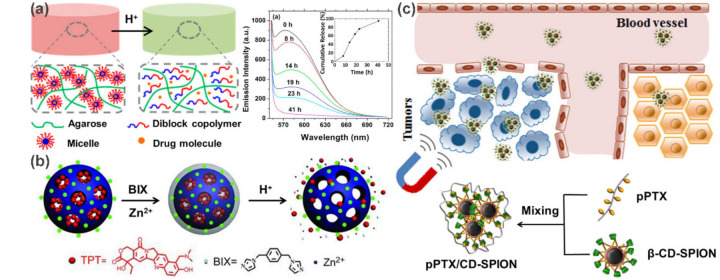
Controlled drug delivery systems for cancer theranostics. (**a**) Agarose hydrogels embedded with pH-responsive micelles for pH-triggered drug release. (**b**) Schematic illustration of the preparation and pH-responsive release of coordination polymer coated MSNs. (**c**) Schematic illustration of the fabrication of pPTX/CD-SPION nano-assemblies and their application in magnetically guided drug delivery. Adapted with permission from Refs. [42,43,46].

For example, Zhao et al. [42] developed a pH-triggered drug release system by incorporating pH-responsive diblock copolymer (PEG–*b*–poly(2-(*N*,*N*-diisopropylamino)ethyl methacrylate) micelles into agarose hydrogels (Figure 4a). The core of the micelle serves as a hydrophobic microenvironment for specific incorporation of hydrophobic drugs, and the subsequent protonation of tertiary ammonia at low pH cause pH-induced dissociation of micelles to achieve ultimately controlled release. Taking Nile Red as a model drug, the fluorescence emission intensity decreased gradually with time when the hybrid gel was treated with a buffer at pH 3.3 until the Nile Red was completely released after 41 h. Another example was reported by Che et al. based on coordination polymer coated MSNs (Figure 4b) [43]. The crucial difference in this work, however, is the pH-responsive characteristic derived from the coordination between polymer layers and MSNs rather than the intrinsic pH sensitivity of the polymer. Similarly, many other types of stimulus-responsive drug delivery systems have been built. In the case of carbon nanotube-based delivery systems, Mandal and coworkers embedded SWCNTs within a crosslinker-free silk hydrogel. Based on the electrical and thermal properties of carbon nanotubes, as well as the hydrogen bonds and electrostatic interactions between SWCNT and DOX, they successively realized the controlled release of DOX in response to pH, heat, NIR light and electric field [44,45].

Targeted drug delivery is an important technique for efficient cancer therapy. Polymer nanocomposites with superparamagnetic characteristics are particularly attractive as they can be directed and localized under the control of an external magnetic field to achieve magnetically targeted drug delivery [32]. For example, an interesting magnetic delivery system was developed by the co-assembly of β-cyclodextrin (β-CD) decorated superparamagnetic iron oxide nanoparticles (β-CD-SPION) and polymerized paclitaxel (pPTX) [46]. As shown in Figure 4c, the obtained nanocomposites (pPTX/CD-SPION) can act as an efficient magnetic nanovector to enrich PTX in tumor regions and significantly inhibit the tumor growth. Clearly, traditional targeting strategies that rely on biologically targeted small molecules (e.g., RGD and folate) are also applicable to the supramolecular polymer nanocomposite systems [44,45,47]. This is even easier to achieve due to the convenience of polymer functionalization.

**Figure 5:**
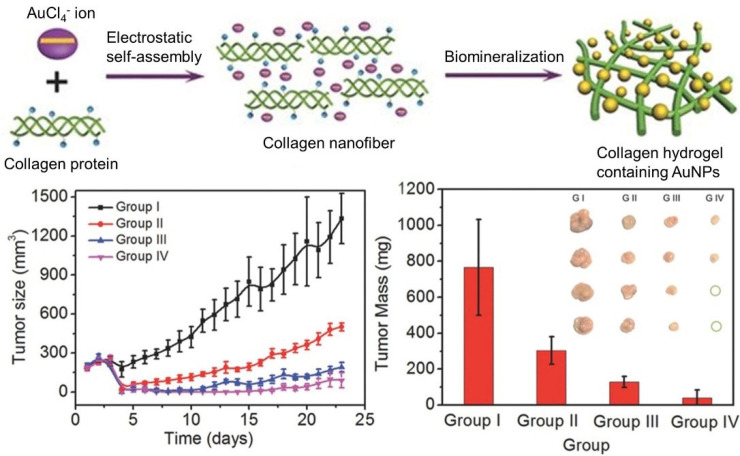
Schematic representation of the formation process of injectable AuNPs-collagen nanocomposite hydrogel via electrostatic self-assembly and biomineralization. Tumor volumes and weights after different treatments demonstrated the synergistic therapy efficacy of PTT/PDT against MCF-7 tumors. Adapted with permission from Ref. [48].

With the deepening of research, multimodal synergistic therapy has attracted more and more attention. Compared to the corresponding monotherapy, a better therapeutic effect can be obtained when more therapies are combined in a specific pattern and sequence to yield the synergistic effects. Supramolecular polymer nanocomposites show distinct advantages in this respect. Yan and coworkers realized the purpose of “one injection, multiple treatments” based on a self-assembling collagen-gold hybrid hydrogel [48]. The collagen hybrid hydrogel was formed by gold-biomineralization-triggered self-assembly (Figure 5). Benefitting from the non-covalent driving forces and the photothermal effect of AuNPs, the formed hydrogel is able to serve as a low-toxic injectable material for both drug delivery and photothermal therapy. Subsequently, the strategy of photodynamic therapy (PDT) was proposed when photosensitive drug such as Meso-Tetra (*N*-methyl-4-pyridyl) porphine tetrachloride (TMPyP) was employed as a model drug. An obvious synergistic therapy efficacy against MCF-7 tumors in mice was observed, as the tumor growth was significantly inhibited and some tumors were even completely eradicated after 23 d of treatment.

### 3.2. Bioimaging

Bioimaging is a powerful technology for visualizing life activities in situ at a cellular or even subcellular level, and plays a vital role in drug tracking and tumor monitoring in the biomedical field. Compared to small molecular probes and conventional polymer probes, the bioimaging probes based on supramolecular polymer nanocomposites often simultaneously possess many important features, such as biocompatibility, target specificity, stimulus responsiveness, and diversity. Specific contrast agents or functional components can be incorporated as desired to prepare diverse bioimaging probes, such as fluorescent components for fluorescence imaging [49,50], paramagnetic components for MRI [51,52], infrared absorbing agents for photoacoustic (PA) imaging [53], and X-ray absorbing agents for computed tomography (CT) imaging [54].

**Figure 6:**
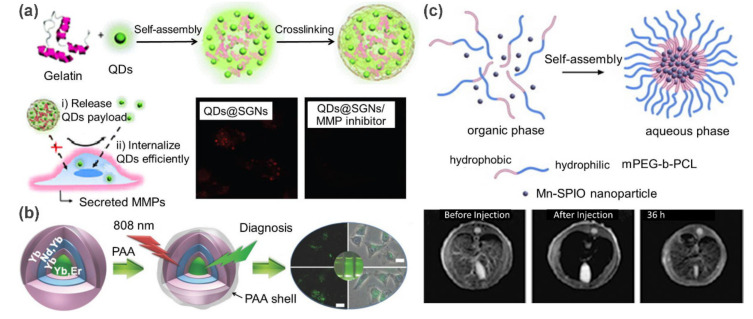
(**a**) Schematic illustration of the preparation of CdSe quantum dots encapsulated supramolecular gelatin nanoparticles. Fluorescent confocal microscopic images demonstrated that QDs were released and further internalized by cancer cells. (**b**) Schematic illustration of the preparation of core-shell UCNP@PAA nanoparticles and their application in fluorescence imaging. (**c**) The formation of hybrid micelles via self-assembly of Mn-doped superparamagnetic iron oxide (Mn-SPIO) nanoparticles and amphiphilic polymer (mPEG–*b*–PCL). MRI imaging results with the Mn-SPIO micelles. Adapted with permission from Refs. [49,50,51].

Currently, QD-reinforced supramolecular polymer nanocomposites are being actively explored for fluorescence imaging. For example, Wang and coworkers [49] prepared a matrix metalloproteinase (MMP) responsive cancer cell fluorescence imaging probe by embedding QDs into supramolecular gelatin nanoparticles (SGNs) (Figure 6a). In this work, a microfluidic platform was employed for the preparation process. By altering the flow rates and physiochemical parameters of components, size-controllable QDs@SGNs were generated. These QDs@SGNs nanocomposites were pre-crosslinked with glutaraldehyde, and thus exhibited sufficient stability. Owing to the sensitivity of SGNs to MMP, this probe exhibits high specificity for tumor cells. In the absence of MMP inhibitors, the fluorescence intensity of cells treated with QDs@SGNs is 1.5 times higher than that in the presence of MMP inhibitors. Additionally, QDs can be released and further internalized to illuminate cells when the gelatin matrix was degraded by MMPs. This work presents a typical proteinase-responsive fluorescence imaging example, in which the QDs@SGNs probes are specifically accumulated at target sites and then self-activate their optical signals. Additionally, UCNPs are widely exploited for fluorescence imaging, in view of their unique upconversion luminescent property. For instance, Lin et al. [50] fabricated a hybrid luminescent probe by coating UCNPs with the self-assembled poly(acrylic acid) (PAA) shells (Figure 6b). A highly efficient upconversion luminescence imaging capacity was observed in the following vitro/in vivo experiments.

Magnetic supramolecular polymer nanocomposites can be used as contrast agents for enhancing MRI. Using Mn-doped superparamagnetic iron oxide (Mn-SPIO) nanoparticles as filler, Ai et al. [51] fabricated an ultrasensitive MRI contrast agent for liver imaging (Figure 6c). In this work, hydrophobic Mn-SPIO nanocrystals were self-assembled into small clusters with the help of amphiphilic methoxy poly(ethylene glycol)–*b*–poly(3-caprolactone) (mPEG–*b*–PCL) and finally formed micellar clustering nanocomposites. In vivo MRI study indicated that T2-weighted signal intensity in liver decreased about 80% at 5 min and the time window for enhanced-MRI lasted up to 36 h after intravenous administration of Mn-SPIO micelles in mice. These advantages make it possible for these nanocomposites to be used for the identification of small liver lesions.

**Figure 7:**
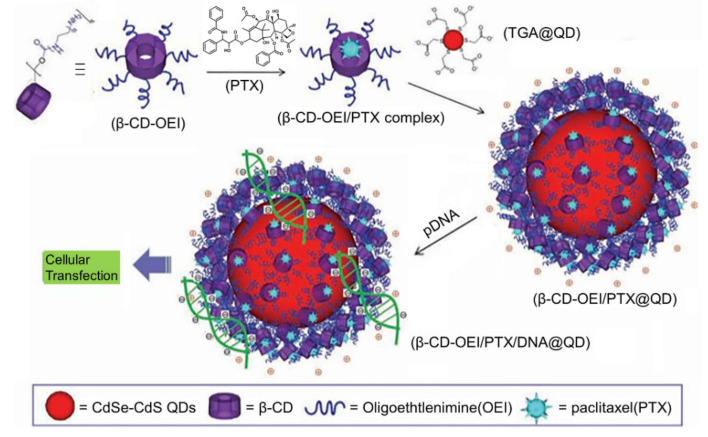
Schematic representation of the fabrication of multifunctional hybrid nanocarrier with simultaneous paclitaxel/gene co-delivery and cellular imaging functions. Adapted with permission from Ref. [55].

Recently, simultaneous drug delivery and bioimaging function for both therapeutic and diagnostic purposes has received much attention because it may achieve better anticancer efficacy. A typical example of such systems was described by Li et al. [55], who constructed a novel multifunctional supramolecular hybrid nanocarrier with synergetic gene and drug co-delivery, and simultaneous cellular imaging function (Figure 7). In this design, a core-shell fluorescent probe (β-CD-OEI@QD) can be prepared by electrostatically coating a red QD with a star-shaped cationic β-CD polymer (β-CD-OEI). Taking advantage of the robust guest binding capacity of β-CD cavity and their positive surfaces, the resultant β-CD-OEI@QD complex is able to function as a carrier for co-delivery of paclitaxel (PTX) and gene. In addition, the fluorescence imaging function allows the localization and tracking of the delivery systems in living cells. Because PTX and the gene behave in a co-operative way, a great enhancement in the gene expression is achieved for efficient cancer therapy. Similarly, Chu et al. [56] reported multifunctional theranostic microcapsules prepared by layer-by-layer self-assembly of magnetite (Fe_3_O_4_) and supramolecular polymers, which display the double functions of pH-responsive MRI, and UV light-responsive drug delivery.

### 3.3. Tissue Engineering

Tissue engineering has emerged as a new field in medical science that aims at creating artificial tissues and organs for graft replacement. This process requires suitable scaffolds to provide 3D support for cell seeding, proliferation, and differentiation. To satisfy the needs of tissue engineering, the designed scaffolds should have the following key characteristics: excellent biocompatibility and biodegradability for in vivo applications; highly porous structures for cellular migration and proliferation; well-matched mechanical strength and elasticity with surrounding tissues; cell–biomaterial interactions; and in the ideal case, the controlled stimulation of the anticipated biological response. Supramolecular polymer nanocomposites are a promising candidate for tissue engineering application in this regard, especially because of their special capability to combine biodegradable polymers (synthetic and natural) with diverse nanofillers. In the past few decades, several bioactive nanomaterials including calcium phosphate, bioactive glasses, silica NPs, nanoclays, and carbon-based nanomaterials were chosen and involved in the construction of composite scaffolds [57,58,59]. Accordingly, numerous bioactive scaffolds based on supramolecular polymer nanocomposites with tailored functionality were generated and have been widely used in both hard (e.g., bone and tooth) [60,61,62] and soft (e.g., cartilage, nerves, muscular, cardiac, skin, and blood vessels) [63,64,65,66] tissues engineering. Meanwhile, a class of fascinating injectable materials emerged [67,68,69]. They are certainly a wise choice for tissue engineering, because they are capable of filling irregular tissue defects in a minimally invasive fashion.

**Figure 8:**
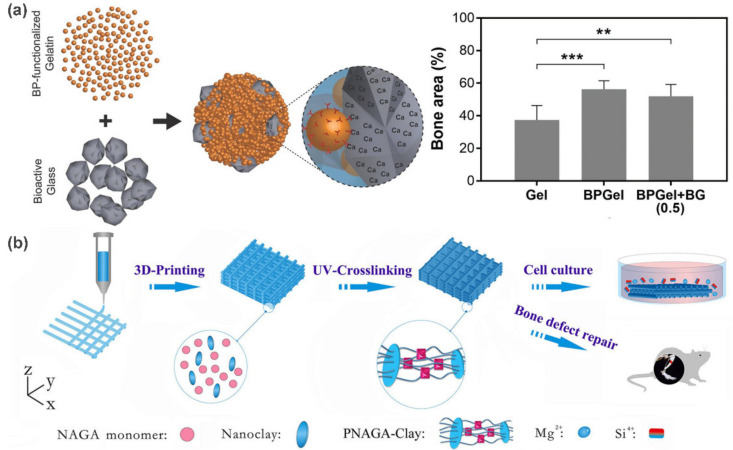
Preparation of bioscaffolds for bone tissue engineering applications. (**a**) Schematic illustration of the formation of injectable composite gels composed of BP-functionalized gelatin and bioactive glass. The increase in bone area confirmed their capacity to support the regeneration of osteoporotic bone defects. (**b**) Schematic illustration of the fabrication process of 3D-printing PNAGA-Clay scaffolds and their application in bone tissue engineering. Adapted with permission from Refs. [60,62].

Bone tissue engineering is an important branch of tissue engineering. Inspired by natural bone, the combination of biodegradable polymers with bioactive ceramic nanoparticles is a typical approach to construct artificial bone engineering scaffolds. As an example, Leeuwenburgh et al. [60] examined the effect of bisphosphonate (BP) and bioactive glass on the regeneration of osteoporotic bone defects. In this work, injectable composite gels were fabricated through the self-assembly of BP-functionalized gelatin and bioactive glass particles (Figure 8a). In vitro experiments showed that the composite gels could induce apatite formation, and facilitate proliferation and differentiation of osteoblastic cells in the absence of exogenous osteogenic supplement. Furthermore, the regenerative capacity was confirmed when more apparent formation of vascularized bone was observed inside femoral condyle defects, suggesting their ability for the treatment of osteoporotic bone defects. Similarly, the self-assembled composite nanoparticle hydrogel that contains chitosan and calcium phosphate was also used for mesenchymal stem cell-based bone tissue engineering [61]. This scaffold can effectively support MSC proliferation in vitro and markedly promoted BMP9-induced bone formation in vivo.

Benefitting from the anisotropic structures and large interacting surfaces, nanoclays are widely used in bone tissue engineering in order to obtain hybrid scaffolds with excellent biocompatibility, enhanced strength, and hierarchical structures. For instance, Liu et al. [62] used 3D printing technology to construct high strength polymer/nanoclay bioscaffolds for bone regeneration (Figure 8b). A hybrid ink with both *N*-acryloyl glycinamide (NAGA) and nanoclay (Laponite XLG) was developed to print PNAGA-nanoclay 3D scaffolds. Remarkably, the final composite scaffolds exhibit superior mechanical performances, in which the tensile strength and Young’s modulus reach 1.17 and 0.18 MPa when the NAGA content is 30%. This can be attributed to the dual interaction networks of H-bonding and nanoclay-polymer chain linkage. In vitro experiments showed that Mg^2+^ and Si^4+^ were continuously released from the PNAGA-Clay scaffold, thus facilitating the osteogenic differentiation of osteoblast cells. Histological observations of implanting PNAGA-Clay scaffold in a rat tibia model showed the formation of abundant new bone around the defect area, which demonstrating their ability for bone regeneration.

**Figure 9:**
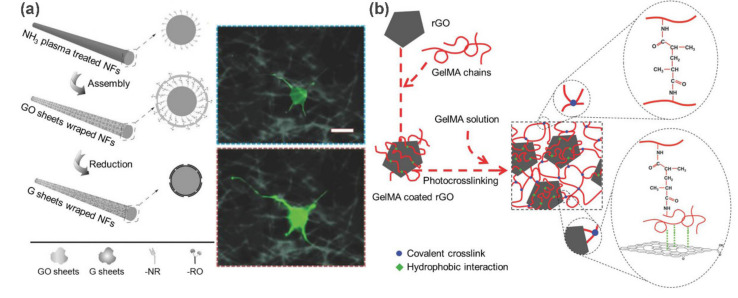
Preparation of electroactive bioscaffolds for soft tissue engineering applications. (**a**) Schematic illustration of the fabrication process of GO reinforced composite nanofibers G-NFs. Fluorescence images of the cells on G-NFs before and after pulse. (**b**) Preparation procedure of rGO-GelMA hybrid hydrogels. Adapted with permission from Refs. [63,64].

In light of their excellent electrical conductivity, carbon-based nanomaterials such as graphene and CNTs can be exploited in the design of electroactive bioscaffolds for neural, muscular, and cardiac tissue engineering [70,71]. Jin et al. [63] reported 3D electrically conductive nanofibers that were fabricated by electrostatically wrapping electrospun polymer nanofibers with GO sheets and followed by a chemical reduction (Figure 9a). As expected, the resulting composite nanofibers (G-NFs) show excellent electrical and mechanical properties. The maximum electrical conductivity reaches 12.5 ± 1.2 S·cm^−1^ and the Young’s modulus (5.3 ± 1.2 GPa) is similar to that of natural collagen fibers. Subsequently, G-NFs were evaluated as a soft cell modulation scaffold for neural tissue engineering, where the growth and development of motor neurons have been significantly accelerated when electrical stimulation (100 mV) was applied. In another example, the rGO was incorporated into gelatin methacrylyol (GelMA) matrix through hydrophobic interactions (Figure 9b) [64]. The resulting composite rGO-GelMA scaffolds not only possess enhanced electrical conductivity and mechanical properties, but also exhibit excellent cell adhesion, proliferation, and maturation. Therefore, rGO-GelMA can be used as conductive hydrogels for cardiac tissue engineering.

**Figure 10:**
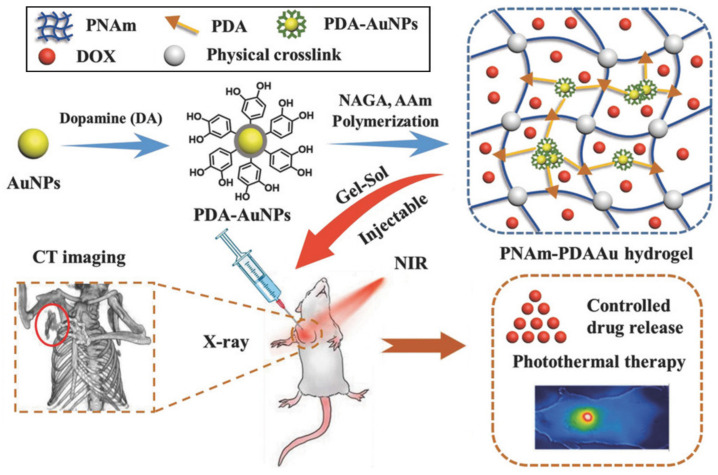
Schematic illustration of the fabrication of PNAm-PDAAu nanocomposite hydrogel and its application as a cancerous breast filler with simultaneous self-imaging and therapeutic functions. Adapted with permission from Ref. [67].

Tissue engineering scaffolds with therapeutic functions are more attractive due to their ability to replace the lesion tissue and to inhibit the recurrence of tumor. As a typical example, Liu et al. [67] reported an injectable supramolecular polymer nanocomposite hydrogel with NIR light-stimulated drug delivery and photothermal therapy for breast reconstruction and breast tumor recurrence inhibition. As shown in Figure 10, This hydrogel was fabricated by entrapping polydopamine coated-gold nanoparticles (PDA-AuNPs) into poly(*N*-acryloyl glycinamide–*co*–acrylamide) (PNAGA-PAAm) hydrogel. Consequently, the hybrid hydrogels not only achieve enhanced mechanical properties due to the covalent and non-covalent interactions between PDA and polymer network, but also exhibit CT self-imaging capacity and superior photothermal effect derived from PDA-AuNPs. The photothermal effect can cause locoregional heating to break the physical crosslinking and further result in a quick gel–sol transition. Therefore, efficient drug delivery can be triggered by the illumination of NIR light. The cumulative DOX release in tumor-like microenvironment was identified to be 82.7%, more than twice the amount of the control groups. Moreover, the compressive moduli of the PNAm-PDAAu hydrogel reaches the same order of magnitude as human breast tissue, showing a great potential for serving as a mammoplastic filler.

## 4. Conclusion and Outlook

This review focuses on the current progress of supramolecular polymer nanocomposites. Based on the huge diversity and versatility of polymers and nanofillers, the combination of the two has greatly enriched the types and functions of nanocomposite materials. Additionally, the introduction of supramolecular chemistry further endows polymer nanocomposites with dynamics and adjustability for various applications, especially biomedicine, as shown in the previous discussion. First of all, the encapsulation of polymer can effectively reduce the possible biotoxicity of nanomaterials to satisfy the basic biocompatibility for biological applications. Secondly, supramolecular polymer nanocomposites are not only versatile carriers for targeted delivery and controlled release of the therapeutic agents, but also perfect candidates for multimode diagnosis and treatment platforms, with the capability of simultaneous multimode synergistic therapy and in vivo imaging. Finally, versatile bioscaffolds for tissue engineering can be rationally constructed by supramolecular polymer nanocomposites with tailored mechanical properties, degradation profiles, and some special bioactives, depicting a bright application prospect for hard and soft tissue repair and regeneration.

Despite significant progress, many challenges still exist. Throughout the developing trend and orientation of supramolecular polymer nanocomposites, in our opinion, there are two key issues that should be addressed in the future. (i) Precise control over structures and properties. Biomedical applications impose strict criteria for such nanocomposite materials, including accuracy in the distribution of nanofillers, as well as the predictability in the acquisition of functions and properties. This will absolutely depend on a deeper understanding of structure–function relationships, as well as the hierarchical assembly strategy similar to nature or more advanced preparation technologies in the future. (ii) Clinical practice. The ultimate goal is to apply them to clinical practice. However, current studies on supramolecular polymer nanocomposites are still preliminary. Moving forward, many crucial problems such as toxicity, biodistribution, biodegradation, and clearance need to be fully evaluated via a large number of in vivo studies before they are applied in clinical diagnosis and treatment. Even so, we are certain that these hurdles will eventually be overcome with the intimate collaboration between scientists in various disciplines.

## Data Availability

The data presented in this study are available on request from the corresponding author.
